# Detoxification Properties of Guanidinylated Chitosan Against Chemical Warfare Agents and Its Application to Military Protective Clothing

**DOI:** 10.3390/polym12071461

**Published:** 2020-06-30

**Authors:** Woong Kwon, Euigyung Jeong

**Affiliations:** Department of Textile System Engineering, Kyungpook National University, Daegu 41566, Korea; kwoong7242@naver.com

**Keywords:** guanidine, guanidinylated chitosan, protective clothes, chemical warfare agent, diisopropylfluorophosphate

## Abstract

This study investigates the detoxification properties of guanidinylated chitosan against chemical warfare agents and its application to the preparation of military protective clothing. Guanidinylated chitosan was synthesized by chitosan guanidinylation with cyanamide. The detoxification properties of the guanidinylated chitosan were then evaluated using a chemical warfare agent simulant, called diisopropylfluorophosphate (DFP). Cotton fabric was treated with 1 wt.% of guanidinylated chitosan in acetic acid and water solution using the simple and conventional textile treatment method of pad–dry–cure. The detoxification properties of the guanidinylated chitosan-treated cotton fabric were evaluated to investigate the application of guanidinylated chitosan to the preparation of military protective clothing. Subsequently, 71.3% of DFP was hydrolyzed to non-hazardous diisopropylhydrogenphosphate (DHP) in 2 h because of the base organocatalytic activity of 0.02 g guanidinylated chitosan itself. Moreover, 60.1% of DFP was hydrolyzed by the catalytic activity of the guanidinylated chitosan-treated cotton fabric, which contained only 0.0002 g of guanidinylated chitosan. This result shows that the guanidinylated chitosan itself has detoxification properties for hydrolyzing DFP to DHP, and its detoxification properties can be more efficient when applied to cotton fabric because it showed 84.3% of the detoxification properties with only 1 wt.% of guanidinylated chitosan. For the first time, this study shows that guanidinylated chitosan has considerable detoxification properties and can be used as an agent to prepare protective clothing.

## 1. Introduction

Protecting the human body against chemical warfare agents (CWAs) is one of the most important functions of military protective clothing. Protection against CWAs can be classified into impermeable and permeable protection. Impermeable protective clothing uses a coating that is impermeable to any liquid containing CWAs, whereas permeable protective clothing utilizes adsorbing materials under regular fabric (e.g., activated carbon), which adsorb liquid to prevent direct contact with the human body [1,2,3,4,5]. Impermeable clothing is not comfortable when worn because it blocks heat, air, and moisture from inside and outside. In contrast, permeable protective clothing is comfortable when worn because it allows heat, air, and moisture to penetrate the fabric [1,2,3,4,5]. However, the adsorbing materials of permeable protective clothing adsorb only limited amounts of CWAs. Furthermore, the protection time duration is limited by the adsorbing capacity of the adsorbent used [4,5]. To overcome these disadvantages, many research projects have studied detoxifying materials that can decompose CWAs into non-hazardous materials and their application to fabrics for preparing protective clothing with detoxification properties [6,7,8,9,10,11,12].

Among the various types of CWAs, organophosphorous nerve agents are the main targets of these studies. Organophosphorus nerve agents inhibit the enzyme acetylcholinesterase (AChE), which catalyzes the breakdown of acetylcholine. The AChE inhibition disrupts neurotransmission, which critically damages the human body [13,14]. Organophosphorus nerve agents include two groups of chemical agents, namely G-agent (e.g., GA, GB, and GD) and V-agent (e.g., VX and VE). Most of these compounds are organic polar compounds based on the P–X structure, where P is phosphorous, and X is fluorine or sulfur. These compounds can be slowly hydrolyzed by water [15,16].

**Figure 1:**
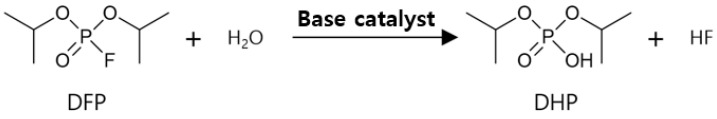
Base-catalyzed hydrolysis of diisopropylfluorophosphate (DFP) to diisopropylhydrogenphosphate (DHP).

Recent reports have shown that various hydrolysis catalysts, such as guanidine, amine, and metal–organic frameworks, can be introduced to prepare fabrics for the hydrolysis of an organophosphorus nerve agent simulant [17,18,19,20]. Instead of actual nerve agents with a lethal toxicity, diisopropylfluorophosphate (DFP) is commonly used in detoxification studies. DFP is converted to diisopropylhydrogenphosphate (DHP) by hydrolysis. This reaction is the general detoxification procedure that can be catalyzed by a base and a metallic catalyst (Figure 1) [17,18,19,20].

Ying et al. reported on the detoxification properties of an electrospun web prepared from the mixture of nylon and synthesized guanidine group-containing polymer with a weight ratio of 1:1. The electrospun web showed 100% DFP hydrolysis to DHP in 2 h at 32 °C [7]. Kim et al. reported on the zirconium hydroxide treatment of electrospun nylon for nerve agent decontamination [8]. Only 40% of DFP was hydrolyzed in 2 h at 32 °C, even when 40% of zirconium hydroxide was treated. Meanwhile, Lee et al. reported on the chemical vapor deposition (CVD) of ethylenediamine, diethylenetriamine, and triethylenetetramine onto the electrospun PAN web for nerve gas detoxification [9]. Consequently, only 10–37% of DFP was hydrolyzed in 2 h at 32 °C, even when the challenging CVD of amine compounds was used. Choi et al. discussed the guanidine group treatment of a thermoplastic polyurethane fiber for nerve agent decontamination. The guanidine-treated thermoplastic polyurethane showed only 50% DFP hydrolysis to DHP in 2 h at 32 °C [10]. In addition, Chen et al. reported on the functionalization of PAN fibers by hydroxylamine hydrochloride for nerve agent decontamination. The functionalized PAN fibers showed that 100% DFP was hydrolyzed after 24 h at room temperature [11]. López-Maya et al. reported on UiO-66 and UiO-66@LiO^t^Bu treatment of silk for the self-detoxification of CWAs. Only 35% of DFP was hydrolyzed in 500 min at room temperature when the silk/UiO-66 used DFP detoxification. The silk/UiO-66@LiO^t^Bu showed only 80% DFP hydrolysis to DHP in 500 min at room temperature [12].

As reported, guanidine groups can be used as organocatalysts because they are a strong base in water (pKa = 13.6) and have antimicrobial properties [21,22,23,24]. Therefore, guanidine-containing compounds are good candidates for preparing military protective clothing with detoxification and antimicrobial properties.

This study proposes the use of guanidinylated chitosan as a detoxification fabric treatment agent. Guanidinylated chitosan is easy to obtain and can be applied to fabrics using a simple and conventional textile treatment method, called pad–dry–cure. In addition, guanidinylated chitosan has good durability during washing because of its low solubility. This study investigates the detoxification properties of guanidinylated chitosan against CWAs and its application to prepare military protective clothing. Guanidinylated chitosan is synthesized by chitosan guanidinylation with cyanamide. The detoxification properties of the guanidinylated chitosan are evaluated using the chemical warfare agent simulant, diisopropylfluorophosphate (DFP). A cotton fabric is treated with guanidinylated chitosan. The detoxification properties of the guanidinylated chitosan-treated cotton fabric were then evaluated to investigate the application of guanidinylated chitosan to prepare military protective clothing. The resulting guanidinylated chitosan-treated cotton fabric exhibits 71.3% DFP hydrolysis to DHP in 2 h at 32 °C, which is a remarkable detoxification property because the amount of guanidinylated chitosan used to treat the cotton fabric is only 1 wt.% of the fabric weight. In addition, the fabric treatment method is only a simple pad–dry–cure process. In summary, this study suggests the simplest and most effective method for preparing military protective clothing.

## 2. Materials and Methods

### 2.1. Materials

Chitosan (deacetylation degree: 77% based on the FT-IR spectrum [25] and 50,000–190,000 Da based on viscosity), cyanamide (99%), and diisopropylfluorophosphate (DFP) were purchased from Sigma-Aldrich (St. Louis, MO, USA). Acetic acid (98%) and sodium hydroxide (93%) were purchased from Duksan Chemical Co., Ltd (Ansan, Korea). Hydrochloric acid (35–37%) and ethyl alcohol (99%) were purchased from Daejung Chemical Co., Ltd (Siheung, Korea).

### 2.2. Chitosan Guanidinylation

**Figure 2:**
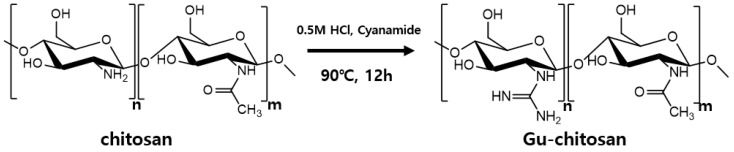
Reaction scheme of the chitosan guanidinylation.

A total of 1.2 g chitosan was dissolved in 50 mL of 0.5-M hydrochloric acid. Subsequently, 3.6 g of cyanamide was added and stirred for 12 h at 90 °C. The mixture was then cooled to room temperature, and 0.5-M sodium hydroxide was added to the mixture for precipitation. The resulted precipitate was washed several times with ethanol to remove unreacted cyanamide and dried at 70 °C in an oven to constant weight. HCl can decrease the hydrolysis of DFP to DHP; hence, HCl in the guanidine group was removed by washing with 1.2-M sodium hydroxide [7]. The resulting guanidinylated chitosan (Gu-chitosan) was dried at 70 °C in an oven to constant weight. Figure 2 illustrates the reaction scheme of the chitosan guanidinylation.

### 2.3. Preparation of the Chitosan and Guanidinylated Chitosan-Treated Cotton Fabrics

**Figure 3:**
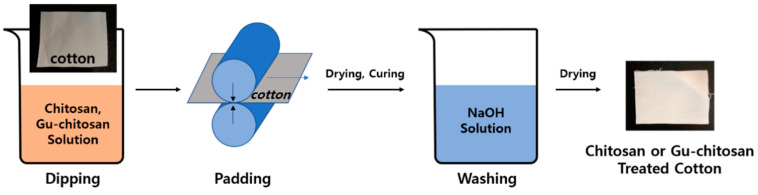
Schematic diagram of the preparation process of the chitosan and Gu-chitosan-treated cotton fabrics.

Chitosan (1 wt.%) or Gu-chitosan (1 wt.%) was dissolved in a 0.17-M acetic acid aqueous solution. The cotton fabrics were dipped into the chitosan or Gu-chitosan solution and padded (100 ± 5% wet pick up) using a padding mangle. The cotton fabrics were then dried for 30 min at 90 °C and cured for 3 min at 140 °C. After which, the chitosan and Gu-chitosan-treated cotton fabrics were washed several times with distilled water and a 1.2-M sodium hydroxide aqueous solution to neutralize acetic acid. The treated cotton fabrics were dried at 90 °C to constant weight. Figure 3 shows the schematic diagram of the preparation process of the chitosan or Gu-chitosan-treated cotton fabrics.

### 2.4. Test of the Detoxification Properties of the Chitosan, Guanidinylated Chitosan, and Prepared Fabrics

A total of 0.02 g chitosan, guanidinylated chitosan, untreated, and treated cotton fabrics (1 cm × 1 cm) was placed into a vial. Subsequently, 0.5 μL DFP and 25 μL distilled water were deposited onto the cotton fabrics and allowed to remain on the fabric for 2 h at 32 °C to hydrolyze DFP. The resulting hydrolysis product (i.e., a mixture of DHP and DFP) was extracted with 800 μL distilled water.

### 2.5. Characterization and Evaluation of the Guanidinylated Chitosan and Prepared Fabrics

The FT-IR spectra of the chitosan, guanidinylated chitosan, and prepared fabrics were obtained using an FT-IR spectrophotometer (Nicolet iS5, Thermo Fisher Scientific., Waltham, MA, USA). The elemental analyses of chitosan and guanidinylated chitosan were performed using an elemental analyzer (Flash 2000, Thermo Fisher Scientific, Waltham, MA, USA). In addition, the X-ray diffraction (XRD) spectra of chitosan and guanidinylated chitosan were obtained using an X-ray diffractometer (EMPYREAN, Malvern PANalytical, Malvern, UK). The XPS spectra of the prepared fabrics were obtained using an X-ray photoelectron spectrometer (K-alpha, Thermo Fisher Scientific, Waltham, MA, USA) with an Al K-alpha source. The X-ray spot size was 400 μm. The surface morphology of the prepared cotton fabrics was investigated with a field-emission scanning electron microscopy instrument (FE-SEM, SU8220, Hitachi, Tokyo, Japan). The extracted mixtures after the DFP hydrolysis test were analyzed through gas chromatography (GC, GC-2030, Shimadzu, Kyoto, Japan) with Elite-1 column (dimethylpolysiloxane, 30 m, 0.25-mm I.D., 0.25 μm, PerkinElmer, Waltham, MA, USA). All GC samples were injected using an autosampler (AOC-20, Shimadzu, Kyoto, Japan) for a reliable quantitative analysis. The extracted mixtures after the DFP hydrolysis were analyzed through ^31^P-NMR (AVANCE III 500, Bruker, Waltham, MA, USA). The ^31^P-NMR samples were extracted with D_2_O instead of distilled water.

## 3. Results and Discussion

### 3.1. Chemical Structure Characterization of the Guanidinylated Chitosan

**Figure 4:**
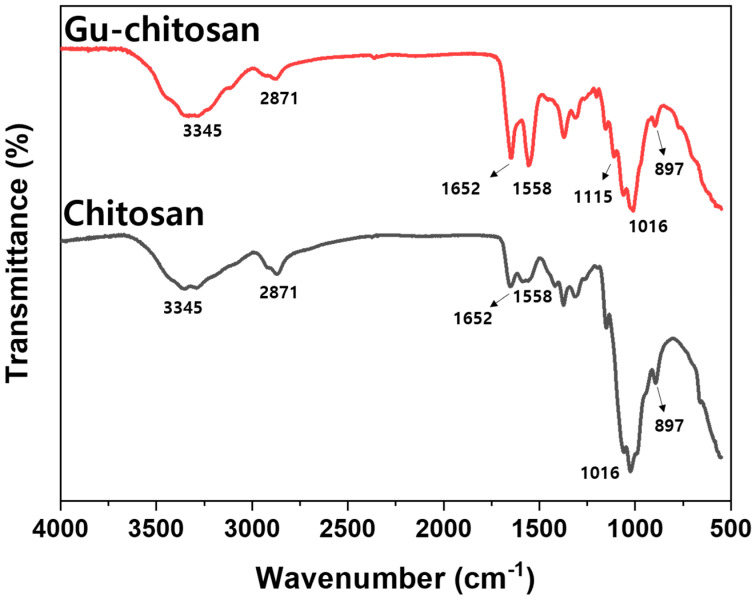
FT-IR spectra of chitosan and Gu-chitosan.

The chitosan guanidinylation was confirmed using FT-IR. Figure 4 shows the FT-IR spectra of chitosan and Gu-chitosan. The broad band at 3345 cm^−1^ in the FT-IR spectra of chitosan and Gu-chitosan was attributed to the NH and OH stretching vibration and the intramolecular hydrogen bonds of chitosan. The peak at 2871 cm^−1^ was attributed to the C–H stretching vibration. Meanwhile, the peaks at 1016 cm^−1^ and 897 cm^−1^ were attributed to the C–O bending vibration and the C–O stretching vibration of chitosan, respectively [25,26,27,28,29]. A new peak appeared at 1115 cm^−1^ in the FT-IR spectrum of Gu-chitosan. Moreover, the peak intensity at 1560 cm^−1^ increased. The new peak was attributed to the C–N–C asymmetric stretching vibration from the guanidine group [27]. The peak at 1560 cm^−1^ was attributed to the –NH_2_ deformation vibration, which can be increased by the guanidinylation of amino groups.

**Figure 5:**
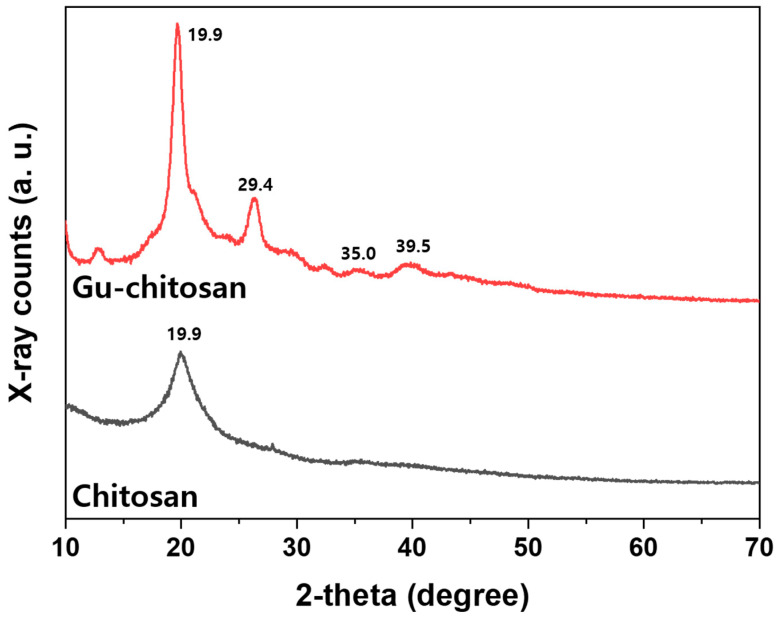
XRD spectra of chitosan and Gu-chitosan.

Figure 5 shows the XRD spectra of chitosan and Gu-chitosan. The XRD spectra of chitosan illustrate the peak at 2θ = 19.9° attributed to the crystalline structure of chitosan. Compared to the chitosan spectrum, new peaks appeared at 2θ = 29.4°, 35.0°, and 39.5° after the chitosan guanidinylation. These peaks are attributed to the introduced guanidine group on chitosan [30]. Therefore, the chitosan guanidinylation was successful, as shown by the FT-IR, elemental analysis, and XRD analysis results.

Table 1 shows the elemental analysis results (C, N, and H) of chitosan and Gu-chitosan and the degree of substitution of Gu-chitosan [27]. After the chitosan guanidinylation, the nitrogen content increased from 7.51 to 10.8, and the atomic ratio of C/N decreased from 5.51 to 3.64. The degree of substitution of Gu-chitosan calculated by the C/N ratio of the elemental analysis was 0.295 [27].

### 3.2. Chemical and Morphological Changes of the Cotton Fabrics Before and After the Treatments

**Figure 6:**
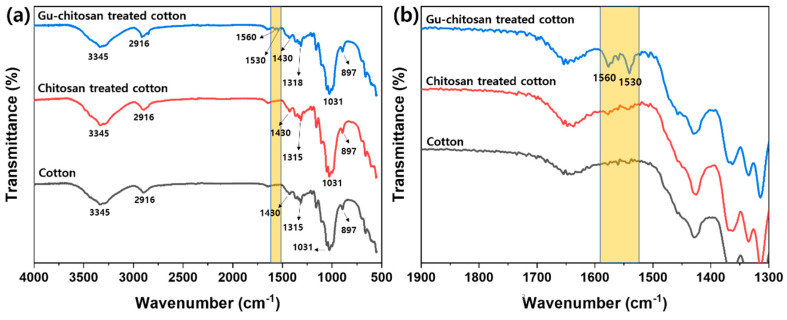
FT-IR spectra of the untreated, chitosan-treated, and Gu-chitosan-treated cotton fabrics: (**a**) FT-IR spectrum in the 4000 cm^−1^ to 500 cm^−1^ range and (**b**) FT-IR spectrum expansion in the 1900 cm^−1^ to 1300 cm^−1^ range.

Figure 6 shows the FT-IR spectra of the untreated, chitosan-treated, and Gu-chitosan-treated cotton fabrics. The broad band at 3345 cm^−1^ in the FT-IR spectra was attributed to the NH and OH stretching vibration and the intramolecular hydrogen bonds of the cotton fabrics. The peaks at 2916 cm^−1^ and 1430 cm^−1^ were attributed to the C–H stretching vibration and the C–H symmetric bending, respectively. Meanwhile, the peaks at 1319 cm^−1^ and 1031 cm^−1^ were attributed to the C–O bending vibration and the C–O stretching vibration, respectively. These peaks appeared in the cotton fabrics [31]. After the cotton fabric treatment with chitosan, compared to the spectrum of the untreated cotton, new peaks attributed to the –NH_2_ deformation vibration appeared at 1530 cm^−1^ and 1560 cm^−1^. This observation suggests that chitosan sufficiently strongly adhered to endure several washing cycles with water and a sodium hydroxide aqueous solution. Peaks at 1530 cm^−1^ and 1560 cm^−1^ appeared after the Gu-chitosan treatment of the cotton fabric. The intensity of these peaks increased compared to that in the spectrum of the chitosan-treated cotton fabric. The peak area of the Gu-chitosan-treated cotton fabric was 11.2, whereas that of the chitosan-treated cotton fabric was 3.8 [32]. In addition, the peak area of the Gu-chitosan-treated cotton fabric was approximately three times higher than that of the chitosan-treated cotton fabrics because the number of the theoretical amine groups of Gu-chitosan was approximately three times higher than that of chitosan.

**Figure 7:**
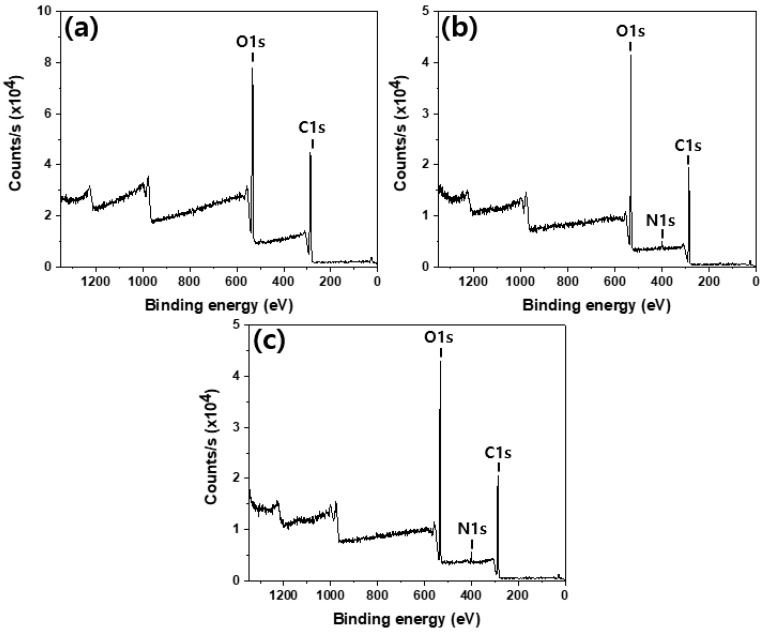
XPS spectra of the prepared fabrics: (**a**) untreated cotton fabric; (**b**) chitosan-treated cotton fabric; and (**c**) Gu-chitosan-treated cotton fabric.

Figure 7 shows the XPS spectra of the untreated, chitosan-treated, and Gu-chitosan-treated cotton fabrics. Only two peaks were observed in the spectrum of the untreated cotton, while three peaks were observed in the spectra of the chitosan and Gu-chitosan-treated cotton fabrics. The new peak at 400 eV was attributed to N1s from nitrogen. The surface atomic concentration was summarized in Table 2 based on the XPS spectra. The surface atomic ratio of N/C increased from 0 to 0.044 after the chitosan treatment and to 0.065 after the Gu-chitosan treatment. The FT-IR analysis results showed that chitosan and Gu-chitosan strongly adhered to the cotton fabric surface, even after several washing cycles.

**Figure 8:**
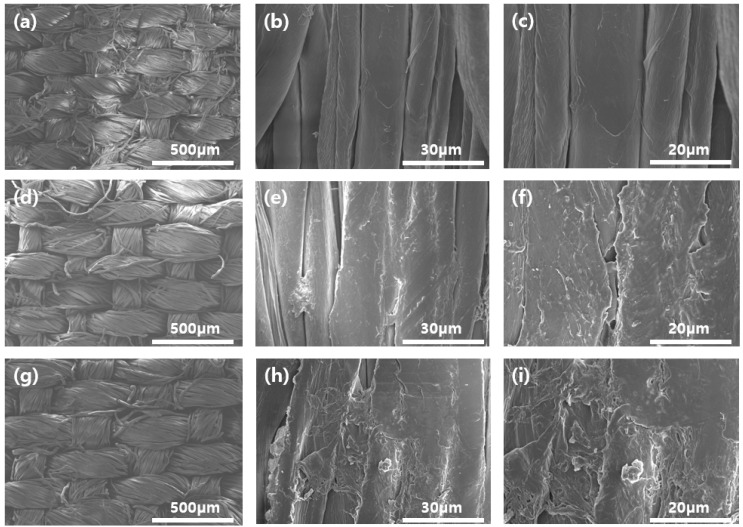
SEM images of the untreated, chitosan-treated, and Gu-chitosan-treated cotton fabrics under different magnifications: (**a**–**c**) untreated cotton fabric; (**d**–**f**) chitosan-treated cotton fabric; and (**g**–**i**) Gu-chitosan-treated cotton fabric.

Figure 8 depicts the SEM images of the untreated, chitosan-treated, and Gu-chitosan-treated cotton fabrics. The untreated cotton fabric surface was fairly clean and smooth. After the chitosan and Gu-chitosan treatments of the cotton fabrics, the spaces between each filament were filled with chitosan or Gu-chitosan, and the fabrics were covered with chitosan and Gu-chitosan [33].

**Figure 9:**
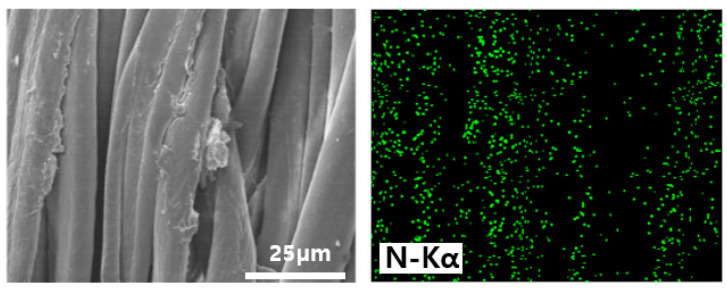
EDS mapping of the nitrogen element for the Gu-chitosan-treated cotton fabric.

Figure 9 shows the EDS mapping of the nitrogen element for the Gu-chitosan-treated cotton fabric surface. The nitrogen distribution on the Gu-chitosan-treated cotton fabric surface was uniform, suggesting that the material was covered with Gu-chitosan. The XPS analysis showed the nitrogen element in the spectrum of the chitosan-treated cotton fabric. In contrast, it was not observed in the EDS mapping analysis. XPS analyzed the area near the surface, which was approximately 10 nm deep from the surface. Meanwhile, EDS analyzed the bulk of approximately 0.5–5 µm depth. Therefore, the portion of nitrogen from chitosan on the cotton fabric surface can be very small compared to that of carbon and oxygen from cotton and chitosan, which resulted in the absence of nitrogen in the EDS analysis [34].

### 3.3. Detoxification Properties of the Prepared Samples

The detoxification properties were evaluated using DFP hydrolysis by the catalytic activity of the chitosan, Gu-chitosan, untreated cotton fabric, chitosan-treated cotton fabric, and Gu-chitosan-treated cotton fabric for 2 h at 32 °C. The DFP hydrolysis amounts were evaluated using the GC analysis. Unfortunately, DHP, which is the hydrolyzed product of DFP, had a much lower volatility than DFP. The boiling point of DHP was higher than the decomposition temperature of the stationary phase of the column used. The DHP peak was not observed under the GC analysis condition herein. The DFP hydrolysis was evaluated by the change in the peak area of DFP. The DFP decontamination ratio can be calculated from the peak area of DFP before the hydrolysis and that of DFP after the hydrolysis. In our previous study, the quantitative analysis of the amount of DFP hydrolysis using only the DFP peak area was sufficiently reliable for use [35].

**Figure 10:**
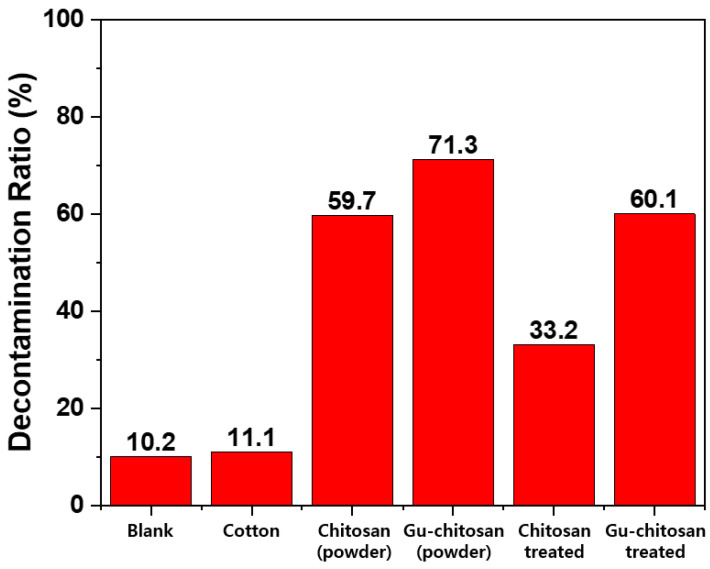
DFP decontamination properties of the prepared samples obtained by GC chromatograms.

Figure 10 shows the DFP decontamination ratios calculated from the GC chromatograms of the extracted samples from testing chitosan, Gu-chitosan, untreated, chitosan-treated, and Gu-chitosan-treated cotton fabric. The decontamination ratio of the untreated cotton fabric was similar to that of the blank sample obtained from DFP in water after 2 h at 32 °C. The DFP decontamination ratios of chitosan and Gu-chitosan were higher than those of the treated cotton fabrics. Chitosan by itself specifically showed double the decontamination ratio of the chitosan-treated cotton fabric. This occurred because the weights of chitosan and Gu-chitosan used were 0.02 g, while the fabric samples measuring 1 cm × 1 cm had weights of 0.02 g, and the amounts of chitosan and Gu-chitosan covering the cotton fabric surface were only 1 wt.% of the fabric weight (i.e., 0.0002 g). Therefore, 60.1% of the decontamination ratio of the Gu-chitosan-treated cotton fabric was very effective as compared to 71.3% of the decontamination ratio of Gu-chitosan by itself. This result was obtained because of the difference in the area of contact with DFP between Gu-chitosan by itself and the Gu-chitosan-treated fabric. When the cotton fabric was treated with Gu-chitosan, Gu-chitosan was spread on the fabric surface as a film, which resulted in a large contact area with DFP. The DFP hydrolysis was catalyzed by the basic property of the guanidine groups. The catalyst with a larger contact with the reactant exhibited a better efficiency. Therefore, even with only 0.0002 g of Gu-chitosan, the Gu-chitosan-treated cotton fabric showed a decontamination ratio similar to that of Gu-chitosan by itself.

**Figure 11:**
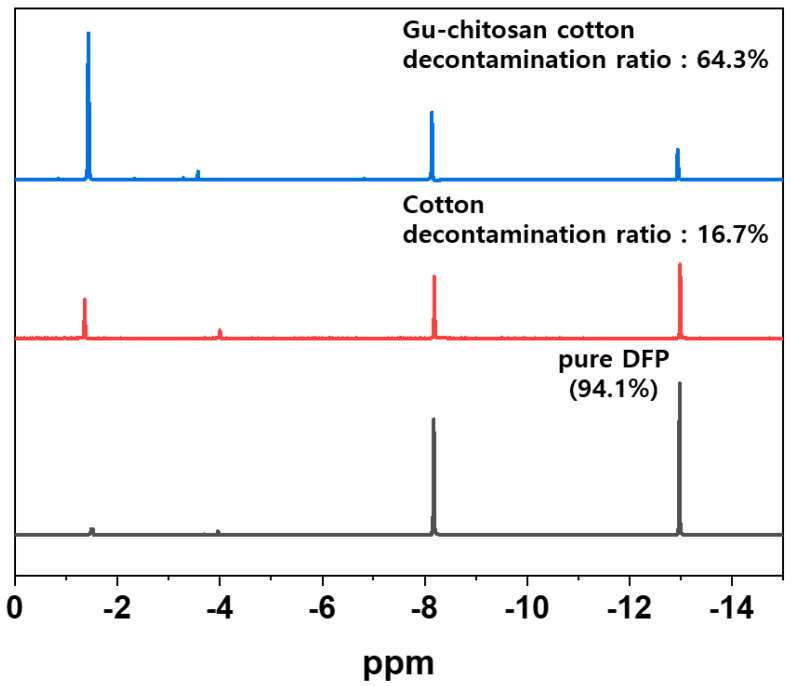
^31^P-NMR spectra of the pure DFP and the extracted DFP and DHP solutions from the untreated and Gu-chitosan-treated cotton fabrics after the decontamination experiments.

Figure 11. shows the ^31^P-NMR spectra of the pure DFP and extracted solutions from the untreated and Gu-chitosan-treated cotton fabrics after the decontamination experiments. The peaks were observed at −8.0 ppm and −13.0 ppm in the spectra of the analyzed samples. These peaks arose because of the P–F bond of DFP. A large peak was observed at −1.3 ppm in the spectrum of the extracted solution from the Gu-chitosan-treated cotton fabrics after the decontamination experiment. This peak arose because of the P–OH bond of the DHP, which was produced from the DFP hydrolysis [36]. The DFP decontamination ratios were calculated by comparing the peak areas of DHP and DFP in the ^31^P-NMR spectra. The DFP decontamination ratios of the untreated and Gu-chitosan-treated cotton fabrics were 16.7% and 64.3%, respectively, which are similar to the DFP decontamination ratios calculated using GC.

On the basis of the DFP decontamination ratios of the Gu-chitosan-treated cotton fabric calculated from GC and ^31^P-NMR for the first time, Gu-chitosan was shown to exhibit excellent detoxification properties along with the Gu-chitosan-treated cotton fabric, even when the amount of Gu-chitosan covering the fabric surface was only 1% of the fabric weight.

## 4. Conclusions

In this study, guanidinylated chitosan was synthesized by chitosan guanidinylation with cyanamide. Moreover, the detoxification properties of the guanidinylated chitosan were evaluated using the chemical warfare agent simulant, DFP. Cotton fabric was treated with 1 wt.% guanidinylated chitosan in acetic acid and water solution using the pad–dry–cure process. Considering 100 wt.% wet pickup, only 1 wt.% of guanidinylated chitosan covered the cotton fabric. The detoxification properties of the guanidinylated chitosan-treated cotton fabric were evaluated to investigate the application of the guanidinylated chitosan to prepare military protective clothing. A total of 71.3% DFP was hydrolyzed to non-hazardous DHP for 2 h because of the base organocatalytic activity of 0.02 g of the guanidinylated chitosan itself. Accordingly, 60.1% DFP was hydrolyzed by the catalytic activity of the guanidinylated chitosan-treated cotton fabric, which contained only 0.0002 g of guanidinylated chitosan. This result shows that the guanidinylated chitosan has detoxification properties to hydrolyze DFP to DHP. Its detoxification properties can be more efficient when applied to cotton fabric because it showed 84.3% detoxification properties with only 1 wt.% guanidinylated chitosan. Therefore, military protective clothing can be prepared by the simple treatment of fabrics with a very small amount of guanidinylated chitosan.

## Figures and Tables

**Table 1:** Elemental analysis and degree of substitution of chitosan and Gu-chitosan.

Sample	Element Content (wt.%)			C/N	DS (Degree of Substitution)
	C	N	H		
**Chitosan**	41.7	7.57	6.65	5.51	
**Gu-chitosan**	39.3	10.8	6.21	3.64	0.295

**Table 2:** Surface atomic concentration of the untreated, chitosan-treated, and Gu-chitosan-treated cotton fabrics.

Sample	Atomic Percent			Atomic Ratios
	C	O	N	N/C
Cotton	62.8	37.2		
Chitosan-treated cotton	57.5	40.5	2.54	0.044
Gu-chitosan-treated cotton	57.5	37.4	3.74	0.065
